# Toledo Medical Association

**Published:** 1879-10

**Authors:** 


### TOLEDO MEDICAL ASSOCIATION.

The 270th Regular Meeting, held Friday evening, Septembea 12th, Vice-President Rigelow in the chair.

*Puerperal Convulsions.*—Dr. Thorn reported the following case: About 5 o'clock Wednesday morning, August 13, I was called from bed by Mr. F., who stated that his wife, who was near her full time, was suffering intense pain of a shooting variety, through the left side of her head, and that this had regularly recurred since the Sunday preceding. I gave two powders each of about quinia, o.32 (gr. v.); morph. sul. o.16 (gr. $\frac{1}{4}$), with instructions to give one immediately and the other in two hours thereafter if neccssary, and promised to visit her about 10 A. M.; by 8:30 I received a note from Mr. F., informing me that one powder had entirely quieted her, that she was sleeping and that it would not be necessary for me to call; 11:30 A.M. I received summons to call immediately, that the patient had had a convulsion. On arrival at the bed-side, I found Mrs. I., an intelligent midwife, who had been engaged to attend at the labor; from her I received the assurance that there had not been a convulsion ; "nothing more than a little nervousness." Neither my examination of the patient nor questions elicited any information that led me to look for convulsions; there was no œdema, no scantiness of urine, no trouble of vision, no pains continuous about the head. Those present or troubling her for a few days, had been recurrent and of a shooting or darting variety and confined to one side of the head, appearing neuralgic in character; as they had subsided, I dismissed them from my mind. But as there were present slight uterine pains, and these had been going on for two or three hours, I made the touch and found parts soft, moist, os well dilated, membranes protruding; these I ruptured, and soon after left the house, though not without telling the midwife and family to send for me if any untoward symptoms should arise. At 2:30 P.M., just as I had entered my carriage to go to the patient, I received the following: "Dear Doctor, our troubles are over; I thank God and you for the delivery, and such a delivery; mother and babe both living and well. I will send for you if necessary." This, I thought, was the end, but about 8 P.M. I received a third note urging me to come immediately, as the patient had had another convulsion. I went without delay and found the midwife at the bedside, calm and not knowing that there had been anything more than "a little nervous restlessness." Pulse soft, 68; no pain, no uneasiness; urine had passed twice since labor, and in good quantity. I remained an hour with patient without seeing anything like convulsions. Ordered

| | | | |
|---|---|---|---|
| B | Potass. Bromid | 8 | ℥ii. |
| | Syrupi Simp | 16 | ʒiii. |
| | Aquæ | 32 | ℥i. |

M. Sig. Two teaspoonfuls every four hours, alternating with elix. valerian. am. in teaspoonful doses; patient not to be disturbed if in a natural sleep. At 8 A.M., August 14, I was again called, this time sent for by the midwife, who met me at the door; she had been with the patient some little time, and had seen her in a convulsion; the nurse reporte eleven during the night; patient hardly out of one before she went into another. Pulse at this time soft, and 78; bed thoroughly wet by escaping urine; mental functions slightly disturbed. The general appearance of patient being so good, she so entirely placid; pulse 78 and soft; skin moist; urine plenty and of natural appearance, (at least that which was seen the night before being so), I did not feel that reccurse to the "lost art" was necessary, and particularly as I had not seen any convulsion. I ordered Norwood's ver. virid o.3.; aqua., o.32. M. Sig. One teaspoonful every hour for two hours, also one teaspoonful from the bromide mixture with 65 centigrams of chloral every hour for three hours alternately with the verat. mixture. While waiting for these prescriptions the patient went into a convulsion, which lasted about seven minutes. During the convulsion, the pulse ran up to 100, hard, face livid, and after convulsion consciousness suspended. These positive evidences decided me as to the necessity of more efficient means, and I immediately had the patient supported in bed in the sitting posture, and drew in a full stream about 768 c. c. of blood; she was then laid on her back; this was hardly accomplished before she began to toss about in convulsive movement, but different from the preceding convulsion. These violent jactications were more like an hysterical or epileptic effort. They lasted about two minutes, and were succeeded by tonic contraction of muscles; congestion of face, the *tout ensemble* of a genuine puerperal convulsion, but of not so great severity or long duration as the preceding one. During the struggle the bandage and compress over the vein were displaced and before knowing it there was a considerable flow of blood, so much so as to thoroughly blanch the face, and to produce sighing respirations, and with this an entire cessation of this and all convulsions. I remained about two hours and took my departure, promising to return at 7 P.M., but did not succeed in seeing her until 10 P.M. At this hour she was quiet; there had been no return of convulsions; ordered some mild nourishment and retired.

August 15, 9 A.M. Patient had passed a quiet and apparently comfortable night, but did not sleep; tongue was soft, clean; pulse 68, soft; ordered generous diet—no medicines. At 9 P.M. all quiet, but no sleep; gave two teaspoonfuls from the bromide and chloral mixture, with orders to repeat this dose every hour for three hours, unless sleep was sooner secured.

August 16, 9 A.M. Patient quiet but had not closed her eyes; continued mixture in teaspoonful doses every two hours. At 9 P.M., patient still wakeful and quiet, but with some little mental purturbation; gave morphia, 22 milligrams hyperdermically and ordered continuance of mixture in teaspoonful doses every two hours until sleep followed.

August 17, 9 A.M, patient had slept soundly all night without more than one dose of the mixture; general condition good, except that mentally she is a little dazed; pulse and temperature normal; skin moist; patient passing urine freely; bowels inactive; ordered potass. et sodæ tartras, 6 grains in half a glass of water every three hours until full catharsis follows; continue generous diet. At 9 P.M. bowels had acted twice or thrice. Discontinued all medication unless patient should be wakeful.

August 18. Patient had a good night without medicine. This morning she seems more rational and disposed to talk. Advised continuance of quiet, and generous diet.

August 19. Patient entirely rational; advised quiet, and good food.

August 21. Patient convalescent. She wanted to know where she had been.

To complete this case, I should state that this confinement was the 14th at full term; all the children born alive, and the first attended with convulsions or other troublesome symptoms. I did not get any chance to test the urine, though I had repeatedly asked the nurse to save it for me; this may be understood when I tell you that at every visit I found a new nurse or helper.

Dr. Forbes—I never hear of these cases without advocating bleeding. Have had about twenty cases of puerperal convulsions in my practice, and have never lost any of them. Have never seen a case where convulsions did not occur previous to delivery; they of course, frequently continue after delivery is accomplishad. I think sufficient blood was not drawn in the case reported, the accidental displacing of the bandage was beneficial. I usually bleed (ad deliquium), frequently cease only when no more blood will flow. Have recently attended a case which illustrates this fact. A lady was taken in labor and soon was seized with a severe convulsion. I bled as soon as possible, about one quart; there was no change in the pulse; she had another convulsion and was bled one quart more; convulsions succeeded rapidly until she had thirteen in all. I bleed her twice in addition, each time a quart, without any appreciable change in the circulation. Twenty leeches were then put to the temples, and the lips became blanched and the pulse was affected; had no more convulsions after this and made a good recovery. I never stop with one bleeding, but continue until this change in the pulse is noticed. I might mention that all my cases were primiparæ excepting one.

Dr. Kirkley—1 am a strong advocate of bleeding. Have had cases where the patient died. One patient had convulsions with the first child; was bled and recovered; again, during her eighth or ninth labor, she had convulsions, and was bled fully and died. Another case I remember, found the patient in convulsions; bled her immediately, and, as soon as possible, delivered her, but she also died.

Dr. Schenetzler asked why the doctor ruptured the membranes and left the case.

Dr. Thorn—I plead guilty of rupturing the membranes and leaving the case. Being pressed with other business, and, as I had full confidence in the midwife who remained, was deceived as to her condition by statements made that there had been no convulsions. This being the fourteenth child, and there having been no convulsions during the previous labors, I did not expect them to occur this time. There were no prodromata to give warning, the only pain she complained of was recurrent and had been relieved by quinia; was also deceived by the condition of the pulse. When called by the messenger, was told that convulsions had occurred, and, therefore, bled her, as given in my report. Also prescribed the veratrum viride and bromide mixture. There are some who believe this treatment is sufficient without venesection. It is not necessary for me to say that I am in favor of blood-letting in these cases.

Dr. Kirkley—When the blood pressure has been great, I believe an organic lesion may take place, even with the first convulsion. When this does occur, nothing will relieve. These cases are exceptional. When the patient does not recover consciousness, I always consider it a grave condition, and the family should be made to understand that in all probability death will insue.

*Summer Diarrhœ.*—Dr. J. T. Lawless reported a case of diarrhœ in a child six weeks old; had been sick since birth; vomits nearly everything taken into the stomach as food; has been unable to retain anything with the exception of Horlick's food. The medication has been opium, bismuth and the alkalies; have kept it under opiates all the time; quinia has done no good; its passages number twelve in the twenty-four hours.

Dr. Chapman—Have used Horlick's food in many cases. Several children have been raised on this alone, where milk or anything else disagreed. It nourishes as well as anything in my experience; have never seen diarrhœ following its continued use. This food is largely composed of malted grain, and I believe, as a rule, these preparations will be well borne. In adults, have had excellent results in cases of mal-nutrition from the use of Trommer's Extract or Reed & Carnrick's Maltine. Dr. Thorn has used malt flour. I would like to hear the result of his experience.

Dr. Thorn—Have used the malt flour with beneficial results. The malted brewer's grain is sifted, and the flour made into gruel. I would recommend Dr. Lawless to follow a line of treatment directed to a proper quality of food, rather than medication. It would be well to freely evacuate the bowels with salines or castor oil.

Dr. Rechnetzler—In such cases, Dr. Jacobi recommends barley water, or barley water and milk.

Dr. Forbes—I believe these cases require management, not medicine; have frequently fed them on barley or rice water alone; it is enough to nourish, especially when feverish. In preparing these foods, they should be boiled four or five hours, keeping up the requisite quantity of water as it evaporates. I use three or four teaspoonfuls of the grain to the pint. Have found excellent results from the use of lactopeptine in ten grain doses, repeted several times a day. I have used maltine frequently with satisfaction in adults, where there was diarrhœ, dysentery and general debility.

Dr. Thorn—In the maltine or extract of malt we get the grape sugar, which I think is advantageous.

Dr. Lawless—I do not think any starch food is good. You must get it in barley or rice water. Horlick's food does not contain starch.

Dr. Chapman—Have used the Swiss condensed milk in several cases, and seen it used by mothers frequently; have had good results from it. Children taking it are not troubled with indigestion, are fat and well nourished.

Dr. Ridenour—I have used considerable Swiss condensed milk in my practice. Have observed a constipating effect following its employment. This is not the case after the child has become accustomed to it. I have never seen a case where it did not agree with the stomach. Don't like the opium treatment; think it interferes with the secretions, and it may cause vomiting. I use and like lactopeptine, if given in large doses. Children who have diarrhæ should have no sugar added to their food.

Dr. Hipp—Malt has no starch. The curd is apt to form in the stomach after constant use of milk. Mares or asses milk is nearer in composition to human milk. I therefore prefer it so obtained.

Dr. Forbes—No one food will agree with all cases. It may be necessary to experiment until the best one is found for a particular patient. I believe the boiling prevents the starch from disagreeing when barley or rice water is used.

Dr. Thorn—In preparing food from condensed or other milk, I think the strength should vary with the case; less water will be better in some cases and more in others. I believe discomfort may be occasioned by the over-distention of the stomach by too much water, a great quantity being taken when the food is too largely diluted, and thereby digestion is interfered with and the child does not get enough nourishment.

Dr. Lawless—Experience shows that in summer complaints of children, opium comes first in point of usefulness. Dr. Smith is authority for saying opium comes first, alkalies second, and astringents third in the list of valuable remedies in these diseases. I think very little sugar should be used when diarrhæ is present.

*Ruptured Perineum.*—Dr. Kirkley reported the following: Was called night before last to a case of labor, os dilating and dilatable, face presentation, pains every five minutes and regular; woman weighing 185 or 190 pounds. The labor was lingering, but towards morning the head pressed upon the perineum, which was rigid. A severe pain coming on, the child passed into the world through a ruptured perineum, the rupture extending from the vulva to, not through, the sphincter ani. I supported the perineum with my hand, but was powerless to prevent the rupture. The cord was three times around the neck of the child, which was dead. To-day I stitched the perineum with three wire stutures. The rupture was, I believe, unavoidable. Why should we sit still and see this accident occur without being able to do anything to prevent it?

Dr. Ridenour—Recommended, as he had done before in the Society, incisions transversely in both labia, and believed this would prevent the accident. The cuts could scarcely be perceived after the labor was accomplished.

Dr. Thorn—I believe Dr. Ridenour's suggestions are good. The rupture in many cases could be prevented in this way.

Dr. Forbes—Benefit might be derived from the application of forceps. By compressing the head it might pass without rupturing the perinem. Have never had but one extensively ruptured perineum in my practic.

Dr. Chapman—I do not believe that in all cases the cutting of the labia as advocated by Dr. Ridenour will prevent the accident. The pressure is frequently toward the rectum. Cases have been reported where the rupture occurred in the perineum without injuring either the sphincter vaginæ or ani. I had in my practice a case of this kind; the patient after a severe labor gave birth to a child through a rent in the perineum. The rupture never gave discomfort. At a second labor the opening was observed, an examination was made both through the vagina and rent. The head was directed by the hand and passed through the proper opening. In such a case cutting the labia could assist nothing, and I do not believe that in any case I would resort to it.

Dr. Kirkley—As to the cutting operation advanced by Dr. Ridenour, I do not believe in it, although I do not entirely condemn it. In the case reported there was not any indication for it; the pressure was backward toward the rectum. The child's head and body were expelled together with a rush.. The forceps could not assist, the pains were efficient, it might possibly have reduced the size of the head, but I think the reresult would have been the same. Have greater confidence in the powers of nature than in any interference by instruments in such cases.

*Foreign Body in the Ear.*—Dr. Curry—About three weeks ago I was consulted by a young Miss, who thought she had a fly in her ear; complained of a constant buzzing and pain; there had been a discharge. On examination the drum-head was inflamed, raw and bleeding. There were seen eight or ten maggots moving about in the meatus. After removing them I found a perforation, which looked fresh, perhaps from the gnawing of the worms. I suppose these worms came from the eggs of a fly which had deposited them there.

Dr. Bigelow—In Texas and some of the Southern States the sand fly frequently deposets its eggs in the ear, and the worms are afterwards hatched. The juice of the datura stramonium will kill them.

*Congestive Chills.*—Dr. Forbes—I was called to see a German farmer, who resided several miles in the country. He had been sick for four or five days with, as was supposed, intermittent fever. I saw him towards evening; found him with a pulse 85 and temperature 101°. Saw nothing to indicate anything but malarial poisoning, as we often see. I prescribed a cathartic and febrifuge; quinia to be given when the fever abated. In two or three hours after my visit he went to sleep and never awoke. I visited him next evening and found a pulse of 140 and temperature between 105° and 106°; pupils normal ; body bathed in profuse perspiration. He died during the night. I have never seen a case of so-called congestive fever. I remember a child whom I treated twenty-five years ago, with symptoms in some respects similar to the one I have reported, to only live four or five hours after my visit. I believe that to be a case of congestive fever. Perhaps the farmer died of the same trouble

Dr. Thorn—It looks to me as if there was some kidney complication in the case reported.

Dr. Bigelow—I can call up case after case such as reported by Dr. Forbes. They were very commonly observed on the Wabash twenty-five or thirty years ago, when I practiced in that country. I call them cases of malarial poisoning, in which the patient is completely overwhelmed by the poison. Depleting these cases does harm rather than good. You must never bleed or purge them; you must rather stimulate. I have frequently given a pint of brandy and sixty grains of quinine during the space of a few hours. Always give quinine before you do anything else.

Dr. Thorn—During the building of the Panama railroad, men were constantly seized with this disease. They would frequently die during the first chill. If enough quinine could be given before the time for the second chill, many would recover.

Dr. Hipp—I had a case of pernicious fever in a man last year, who died half an hour after I had seen him. The history of the case was similar to that of malarial fever.

Dr. Ridenour—I have seen congestive chills in Toledo. I first saw them on the Kanawha river during the war. The first case I saw I did not know the disease. I found a man collapsed, circulation at a stand still, and almost comatose. I attempted to bleed him, but could get no blood; he afterwards became completely comatose and died. A day or two afterward I saw another case; gave five grains of quinine every hour; with the first dose a teaspoonful of laudanum, which I afterward continued in twenty drop doses when I gave the quinine. I like opium better than brandy as a stimulant in these cases. Patients are apt to die in the second chill. We have an expression of the congestive chill frequently in the convulsions of infancy. This convulsion takes the place of the chill. They may develop into coma. If the convulsions are interrupted and quinine is given freely, they will get well. Have seen congestive chills passing into coma cured by quinine. The disease is simply a malarial fever of severe degree. If in an adult, you find at the beginning of the sickness a temperature of 105° or 106°, you may be almost certain you have intermittent fever.

Dr. Curry—I have had experience in this disease; think the manifestations vary in different cases. I was once called to see a man who was taken with a chill. Before I saw him another messenger came, saying he was passing blood in large quantities from the bowels. Authors say this is one of the manifestations of the disease. I found him with headache and other symptoms of malarial poisoning, and gave him quinine. I believe the dose of quinine varies with the patient. I remember one who required sixty grains before any effect was observed.

Dr. Forbes—I would mention that a daughter of the farmer was lying in the same room, with as nearly the same conditions as her father, as is possible to see. I certainly did not observe any difference. She recovered with the same treatment prescribed for the other patient. They told me that the fever never left the man, but began to rise after I left, and, as I have reported, he never became conscious.—*Toledo Medical and Surgical Journal.*

